# VSLAM method based on object detection in dynamic environments

**DOI:** 10.3389/fnbot.2022.990453

**Published:** 2022-09-02

**Authors:** Jia Liu, Qiyao Gu, Dapeng Chen, Dong Yan

**Affiliations:** School of Automation, C-IMER, B-DAT, CICAEET, Nanjing University of Information Science & Technology, Nanjing, China

**Keywords:** dynamic target detection, VSLAM, YOLOv3, GMM, Kalman filter

## Abstract

Augmented Reality Registration field now requires improved SLAM systems to adapt to more complex and highly dynamic environments. The commonly used VSLAM algorithm has problems such as excessive pose estimation errors and easy loss of camera tracking in dynamic scenes. To solve these problems, we propose a real-time tracking and mapping method based on GMM combined with YOLOv3. The method utilizes the ORB-SLAM2 system framework and improves its tracking thread. It combines the affine transformation matrix to correct the front and back frames, and employs GMM to model the background image and segment the foreground dynamic region. Then, the obtained dynamic region is sent to the YOLO detector to find the possible dynamic target. It uses the improved Kalman filter algorithm to predict and track the detected dynamic objects in the tracking stage. Before building a map, the method filters the feature points detected in the current frame and eliminates dynamic feature points. Finally, we validate the proposed method using the TUM dataset and conduct real-time Augmented Reality Registration experiments in a dynamic environment. The results show that the method proposed in this paper is more robust under dynamic datasets and can register virtual objects stably and in real time.

## 1. Introduction

Initially, SLAM (Simultaneous Localization and Mapping) was proposed to solve the problem of robot movement in an unknown environment. After the robot observes the environment, it immediately feeds back its posture and movement trajectory, and constructs a map of the environment simultaneously. The early SLAM system mainly used single-line lidar, sonar and other sensors to realize its own positioning. With the rapid development of computer vision, the VSLAM (Visual SLAM) system with the help of cameras has begun to become the mainstream of research by various teams due to its convenient use and low cost. The VSLAM system has been well applied in the fields of augmented reality (Calloway and Megherbi, 2020), driverless driving (Nguyen et al., 2018), and robotics (Liu, 2021). Virtual objects registered with VSLAM technology have better stability and accuracy in today's popular augmented reality applications. To achieve a more immersive visual experience in the dynamic environment of mobile devices, the VSLAM system with AR (Augmented Reality) technology needs a more excellent background update mode.

Many Augmented Reality Registration methods are based on the front-end visual odometry of SLAM systems, while many VSLAM systems are usually built on static environments. However, the real environment is much more complicated than the ideal environment. Dynamic objects such as people and cars are often unavoidable in scenarios such as classrooms, hospitals, and outdoor shopping places. Those VSLAM systems built on a static environment have poor adaptability to dynamic and complex scenes, leading to substantial errors in the obtained map points and pose matrix (Cheng et al., 2019). Indirectly, it will cause problems such as drift of virtual objects registered in the world coordinate system. Aiming at the problems of excessive pose estimation error and easy loss of camera tracking in the commonly used VSLAM algorithm in high dynamic scenes, we propose a real-time tracking and mapping method based on GMM combined with YOLOv3. This method can guarantee the robust registration of virtual objects in dynamic environments.

To ensure that the camera produces robust results when moving, we combine the affine transformation matrix to correct the continuous frame image (Sun et al., 2022). In the non-key frame stage, we employ GMM (Gaussian Mixture Model; Stauffer and Grimson, 1999) to model the background image, effectively utilizes the global discontinuity of the keyframe, and increases the GMM training time to improve the training effect of the background model. When creating the keyframe, we combine the image frame of the previous time series to segment the foreground dynamic area, and provide prior knowledge for the YOLO detector. To improve the detection accuracy of the dynamic target of VSLAM, we employ the observation value provided by YOLO (You Only Look Once) v3 (Redmon and Farhadi, 2018) in the tracking thread to predict the area of the dynamic target in real time. Our method combines the dynamic area detected by YOLO with the dynamic area obtained after GMM training, and uses the IOU (Intersection Over Union) result as the probability information to obtain the largest possible dynamic target. We choose YOLOv3 because it is a single-stage detector that can achieve good accuracy while meeting the real-time nature of Augmented Reality Registration. Moreover, compared with traditional methods such as frame difference method, optical flow method, and background removal method, YOLOv3 has better real-time performance and robustness. But the disadvantage is that YOLOv3 does not provide prior knowledge that can identify dynamic regions. Our method is complementary to both. It uses a GMM model to train background images, estimates motion regions when creating new keyframes, and provides priors for YOLOv3. At the same time, YOLOv3 meets the real-time and robustness requirements, and can achieve dynamic target detection between consecutive frames.

**Figure 1:**
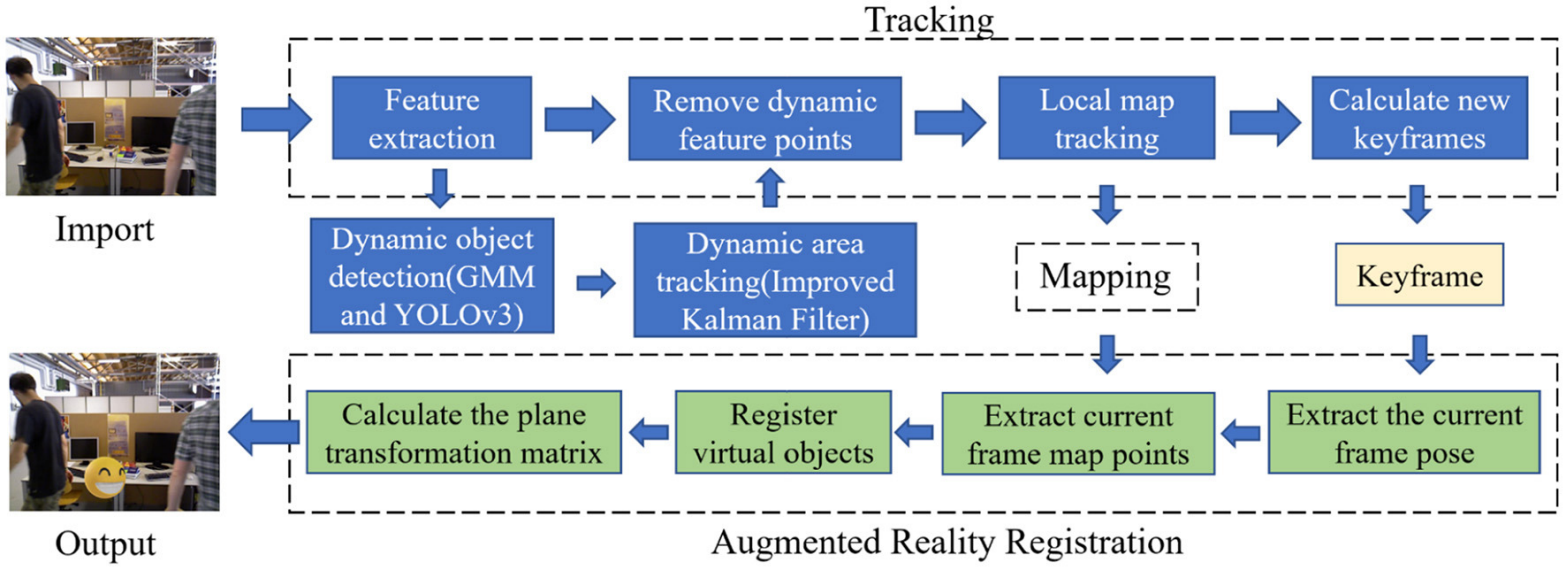
System diagram. This system mainly consists of three parts: tracking thread, mapping thread, and Augmented Reality Registration thread. The tracking thread uses GMM combined with the YOLOv3 method for dynamic object detection and uses the improved Kalman filter method for dynamic object tracking. Next, it removes the feature points of dynamic objects in the keyframes and transfers the keyframes that retain the static feature points to the augmented reality registration thread. In the Augmented Reality Registration thread, it registers virtual objects through the map points and camera poses of the current frame and tracks virtual objects through matrix calculation.

The most traditional tracking algorithm is the filtering algorithm based on the Bayesian framework (Goan and Fookes, 2020). It utilizes prior information to make an optimal estimation of the state of the target in the current frame to track the target, such as the Kalman filter (Xu Y. et al., 2018) and the Particle filter (Chakravarty et al., 2017). In actual operation, the observed value is easily affected by factors such as the camera itself and lighting. The traditional Kalman filter will affect the next predicted value when an error occurs in the observed value, leading to the accumulation of errors. We provide an improved Kalman filter method that uses the first N groups of observations to establish a nonlinear fitting curve to predict the next set of observations. Then, we employ an evaluation metric to determine whether to choose the predicted “observed value” or the value observed by the system. After the update, it can obtain a more accurate and practical background. We employ this improved Kalman filter algorithm to predict and track YOLO objects to ensure the continuity of the regional frame. The real-time accuracy of background map construction determines the reliability of VSLAM applications in many directions. This method can accurately eliminate the dynamic noise during the mapping thread, and obtain a good mapping effect, providing a good mapping environment for Augmented Reality Registration. The system diagram is shown in Figure 1.

The rest of this paper is structured as follows. The second section describes the related work of VSLAM implementation in a dynamic environment. The third section describes the method of this paper in detail. The fourth section gives the experimental results. The fifth section gives some conclusions and analysis of the experiment.

## 2. Related work

### 2.1. Classic VSLAM

The classic VSLAM system has gone through a series of explorations and improvements and has formed an effective execution framework. Davison et al. (2007) first proposed MonoSLAM, a SLAM scheme based on a monocular camera. Klein and Murray (2007) proposed a keyframe mechanism in the PTAM scheme, which realized the parallelization of tracking and mapping, distinguished the front and back ends for the first time, and used nonlinear optimization as the back-end optimization scheme. The two earliest proposed VSLAM solutions have problems such as small application scenarios and easy tracking loss. However, these innovative framework ideas have been used to this day.

Subsequently, scholars began to improve the front-end visual odometer. At present, the feature point method composed of key points and descriptors is the most mainstream front-end algorithm, such as SIFT (Lowe, 2004), SURF (Bay et al., 2006), and ORB (Rublee et al., 2011). This kind of method is stable and relatively mature. Another front-end algorithm is the direct method based on pixel brightness information. Engel et al. (2014, 2015) proposed LSD-SLAM, which uses the direct method to build maps. It has the advantages of fast speed and good real-time performance, but it is very sensitive to the camera's internal parameters and exposure, and it is easy to lose when the camera moves quickly. Forster et al. (2014) proposed high-speed real-time mapping using the sparse direct method SVO. It is extremely fast, but due to abandoning the calculation of the descriptor, its pose estimation is prone to cumulative errors. When the camera moves quickly, the location information is easy to lose, and it is difficult to relocate after being lost. In the case of much noise in the dynamic environment, the result of this method is still not satisfactory.

Mur-Artal et al. (2015) proposed a monocular ORB-SLAM system. ORB-SLAM utilizes unified ORB features in each link of tracking, mapping, relocation and loop detection. It has high computational efficiency, good rotation and scaling invariance (Mur-Artal and Tardós, 2017; Campos et al., 2021), and its performance in a dynamic environment can be further improved. Many SLAM systems improved through dynamic target detection and deep learning are also implemented under the ORB-SLAM's framework.

### 2.2. Dynamic VSLAM scheme of deep learning and geometric view

In terms of dynamic target detection, traditional methods are greatly affected by scene brightness changes, noise, etc., and there will be false detections and missed detections in the target detection process. This also leads to drift during target tracking, which in turn affects the accuracy of target tracking (Huang et al., 2022).

In recent years, dynamic target detection has put forward higher tracking accuracy and target number requirements, and many excellent SLAM frameworks have emerged continuously (Gehrmann et al., 2019). In the past, semantic segmentation was used to train static objects to generate semantic maps that increase the amount of information. For example, the semantic map construction proposed by Goerke and Braun (2009). When building a map in a dynamic environment, it is necessary to segment and remove dynamic characters. The method proposed by Wang et al. (2016) is a new method for classifying human motion regions. It divides human activities into categories and predicts the travel of the human body through general movement patterns. But this method is only suitable for fixed cameras. Riazuelo et al. (2017) proposed a semantic SLAM method in dense portrait scenes. This method solves the limitation of camera fixation. It completes a complete SLAM system based on the visual odometer (Wang et al., 2007). It can detect which are dynamic objects, but it cannot detect changes caused by static objects. Bescos et al. (2018) proposed DynaSLAM, which employs the Mask RCNN (Ammirato and Berg, 2019) to arrange the scene prior knowledge and estimate the possible moving targets through the geometric view method. This method removes the feature points of the moving target through a mask to maintain the algorithm's accuracy. After removing the dynamic target, the previously observed static information is used to repair the area. However, when repairing the occluded background of the current frame, using the pixel area corresponding to the last frame will cause the accumulation of errors. Zhong et al. (2018) proposed Detect-SLAM. It combines the single shot multibox detector (Liu et al., 2016) on the basis of ORB-SLAM, and uses semantic information to eliminate the influence of dynamic targets in SLAM. In addition, it also contains a method to propagate the dynamic possibilities of each feature point in real time, which solves the problem of delay in the transmission of semantic information. Yu et al. (2018) proposed DS-SLAM, which utilizes the optical flow method to track feature points and employs RANSAC (Raguram et al., 2012) to eliminate outliers and calculate the basic matrix. The dynamic and static points are judged based on the distance from the feature point to the epipolar line. Then, SegNet (Badrinarayanan et al., 2017) is used to divide the dynamic area and eliminate the feature points of the dynamic area. However, because the semantic information is not comprehensive enough and the semantics are untargeted, there are problems in dynamic filtering in some aspects, such as gesture occlusion. Xiao et al. (2019) proposed Dynamic-SLAM. Based on the same work as DynaSLAM (Bescos et al., 2018), it reduces the dynamic error and builds a better map (Fan et al., 2020).

These SLAM systems for dynamic scenes generally use semantic information, either using geometric information or a simple combination of methods for dynamic object detection. Cui and Ma (2019) proposed SOF-SLAM, which combines semantics and optical flow methods. It fully utilizes the dynamic characteristics of features hidden in semantic and geometric information. Cui and Ma (2020) proposed SDF-SLAM, utilizes a depth filter to describe each map point's inverse depth, updates the inverse depth of the 3D map points in the Bayesian framework, and divides the 3D map points into active or inactive points. However, the problem of using a semantic combination of VSLAM is still undeniable. They all rely heavily on the training effect of the network model. The prestage workload is enormous, but it can only be divided and cannot be tracked well. For the classic network model of target detection, the candidate area method proposed by RCNN is very time-consuming and cannot be run in real time. YOLO innovatively proposed merging the candidate area and recognition process in RCNN to increase computing speed significantly (Redmon and Farhadi, 2018).

Inspired by deep learning, improved view geometry methods are also constantly advancing, and new system models appear. Sun et al. (2017) proposed a motion removal method based on RGB-D cameras. Since this method relies on the maximum posterior scheme to determine the foreground, the segmentation results are limited (Sun et al., 2019). Xu X. et al. (2018) proposed a multi-view spectral clustering framework that combines multiple models together, integrating the affine, tomography, and basic matrix. Sun et al. (2018) proposed MR-SLAM, which improved their previous method (Sun et al., 2017) to model prospects in different classes, so the number of moving objects was not limited during segmentation. This method adds online learning capabilities, allowing it to update the foreground model incrementally. Although, MR-SLAM can effectively deal with dynamic factors, it consumes too much time in the process of precise detection and segmentation of moving targets, and it is not outstanding in real-time performance. Cheng et al. (2019) inspired by deep neural networks, proposed SMR-SLAM, which employs the Bayesian formula to solve the probability distribution of the feature point area of the geometric view. Small-probability events are eliminated to help SLAM distinguish dynamic regions as much as possible. It can learn and perform well in scenes with low dynamics, but the error is more evident in highly dynamic scenes or excessive complexity. Liu et al. (2022) optimized the sparse point cloud map through the YOLOv4 framework to enhance the interactivity of the robot. Gao et al. (2020) proposed a feature map fusion one-shot multi-box detector, which has higher detection accuracy and real-time performance compared to SSD and DSSD methods. The occlusion of the hand is also one of the reasons for the failure of virtual object registration, and the detection of the hand is also very necessary (Gao et al., 2019). YOLOv3 has better real-time and accuracy in hand detection, and can detect hand occlusion in real time, helping to complete better virtual object registration.

We provide the method of GMM combined with YOLOv3. Our method uses the ORB-SLAM2 framework and improves its tracking thread to analyze dynamic targets. The method provided in this paper detects and tracks dynamic targets, eliminates dynamic points in real time, and optimizes the update mode of the background to ensure the accuracy of pose solving and map creation.

## 3. Method

### 3.1. Dynamic target detection

#### 3.1.1. Moving target detection algorithm based on GMM

GMM is a method to accurately quantify things with a Gaussian probability density function and decompose them into several models based on a Gaussian probability density function. The GMM application in background elimination establishes a Gaussian mixture model for each pixel in the video frame. If the pixel model has a significant weight, it is indicated as a background pixel; otherwise, it is a foreground image. Since background pixels often occupy high weights, the generated data is more trustworthy on the background pixels so that GMM can distinguish the foreground and the background in the long-term observation sequence generated by the video.

Our method employs the ORB feature extraction and matching to extract feature points, calculates the affine matrix M by matching the front and back frames, corrects the current frame image through the affine matrix M. Then, the method employs GMM to learn background pixels and segment the foreground and background images by finding the pixel group closest to the background. The dynamic target detection process is as follows:

a. In the initialization phase, the method completes the initial setting of the parameters of the GMM model.
b. In the non-key frame stage, the feature points are extracted from the VSLAM to match the continuous frame images. Calculate the affine transformation matrix M by this method, utilize the M matrix to make affine changes, and set the threshold (the threshold is 20 in this paper) to correct the current frame.
c. At the same time, to reduce the image shift caused by the affine matrix error, the method uses the mean filter to process the images before and after the transformation.
d. Our method trains the corrected image on the GMM Gaussian mixture model. It combines the image frame of the previous time series to determine the foreground dynamic area when the keyframe is created.

**Figure 2:**
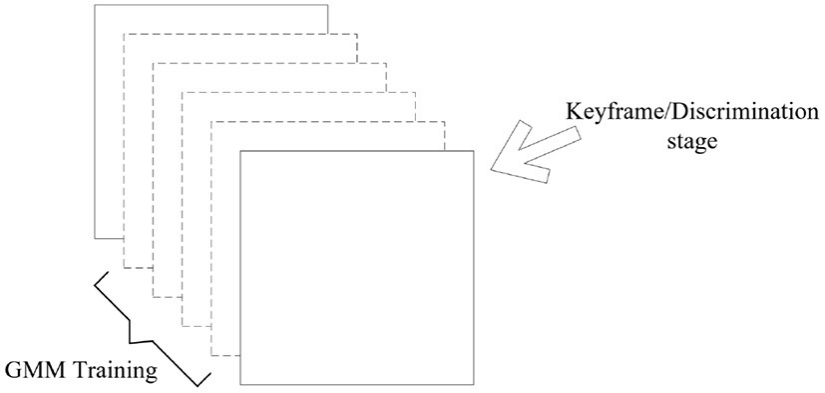
GMM dynamic solution.

**Figure 3:**
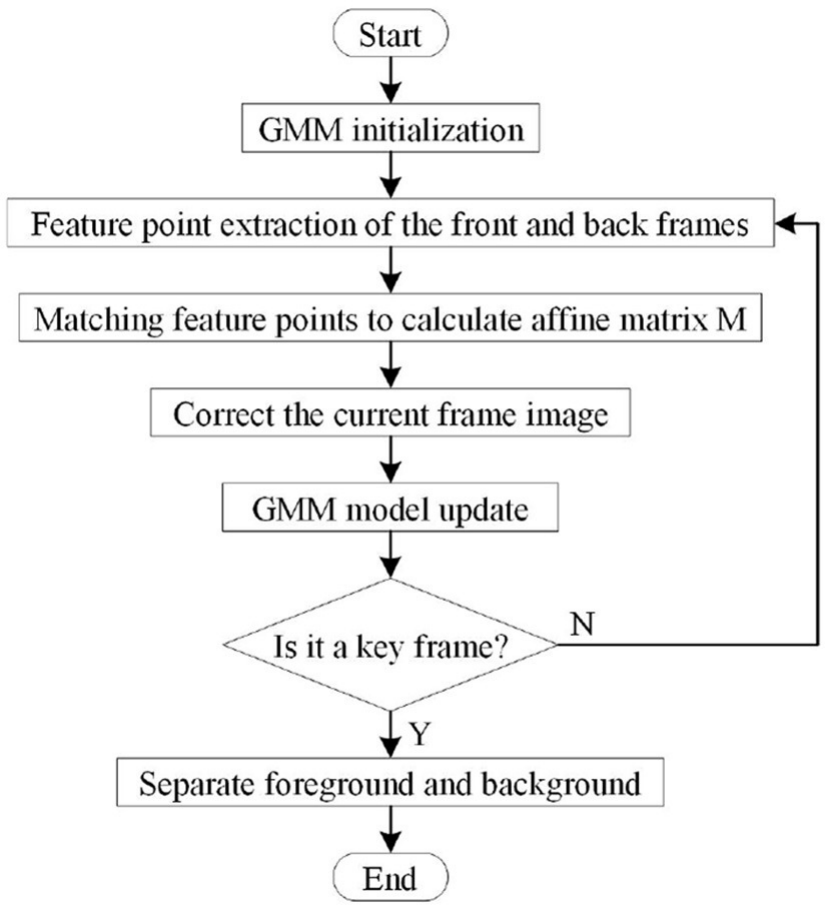
Flow chart of dynamic target detection combining GMM and affine matrix.

The principle diagram of dynamic target detection combining VSLAM and GMM is shown in Figure 2, and the specific flow chart is shown in Figure 3.

#### 3.1.2. Target detection algorithm based on YOLOv3

**Figure 4:**
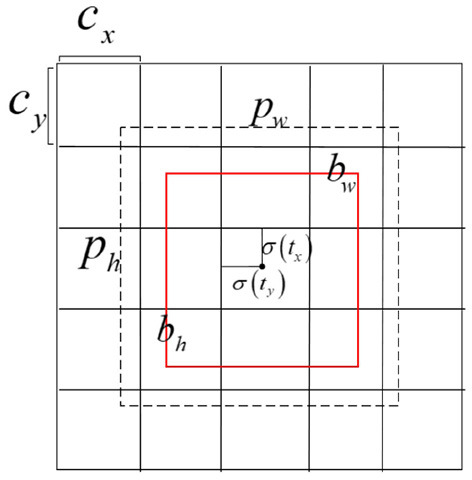
Schematic diagram of bounding box regression.

The moving target detection algorithm based on the GMM extracts the keyframes and then performs the difference. Between keyframes, we employ the YOLOv3 algorithm to detect objects that may need to be tracked. YOLOv3 is a single-stage detector that can meet real-time performance for Augmented Reality Registration while maintaining accuracy compared to methods *via* R-CNN. The method divides the input image into a 13×13 table and then lets each cell detect the target. The bounding box and the discrimination probability value through each grid are obtained to judge whether the target object and the position information and probability information of the target area in the grid. The dimensional clustering method on the bounding box is chosen to select 3 scales and nine types of bounding boxes, the bounding box detection problem is converted into a regression problem, and the 4 coordinates *t*_*x*_, *t*_*y*_, *t*_*w*_, *t*_*h*_ (as shown in formulas 1–4) of each bounding box are predicted. For the problem of bounding box regression, for the 13×13 feature scale map, we utilize three bounding boxes of 10×13, 16×30, and 33×23 pixels; for the 26×26 feature scale map, we utilize three bounding boxes of 30×61, 62×45, 59×119 pixels; for the 52×52 feature scale map, we utilize three bounding boxes of 116×90, 156×198, 373×326 pixels. The regression diagram of the bounding box is shown in Figure 4.

**Figure 5:**
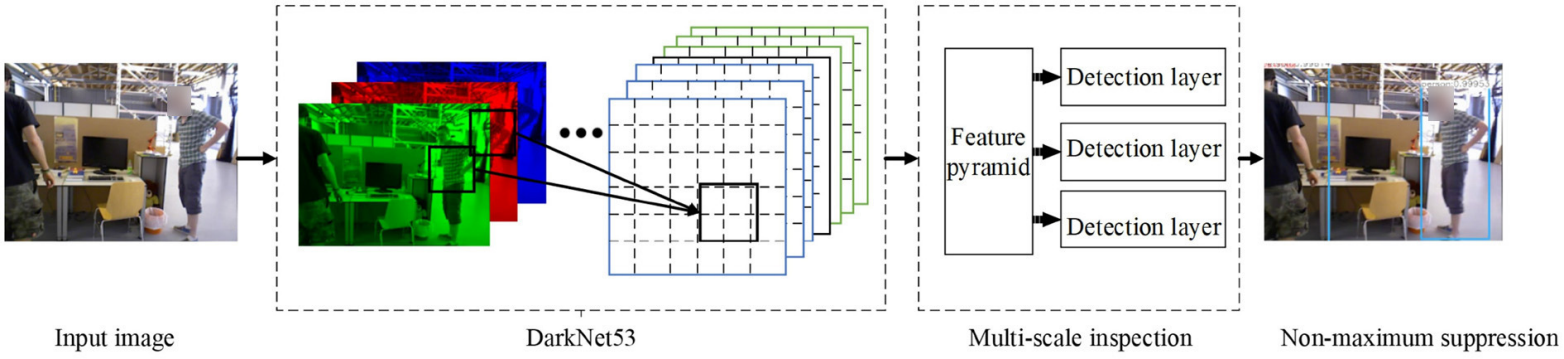
YOLOv3 detection process.

The definition formula for the bounding box is as follows:


(1) $$\begin{array}{l} b_{x}=\sigma \left(t_{x}\right)+c_{x} \end{array}$$

(2) $$\begin{array}{l} b_{y}=\sigma \left(t_{y}\right)+c_{y} \end{array}$$

(3) $$\begin{array}{l} b_{w}=p_{w}e^{t_{w}} \end{array}$$

(4) $$\begin{array}{l} b_{h}=p_{h}e^{t_{h}} \end{array}$$

where *t*_*x*_, *t*_*y*_, *t*_*w*_, and *t*_*h*_ represent the offset of x coordinate, y coordinate, width, and height offset, respectively. *b*_*x*_, *b*_*y*_, *b*_*w*_, and *b*_*h*_ represent the result of the final goal box. σ(*x*) represents the Sigmoid function. The result of x is normalized to speed up network convergence, where *p*_*w*_ and *p*_*h*_ are the width and height of the bounding box, respectively. The overall YOLOv3 detection process is shown in Figure 5.

**Figure 6:**
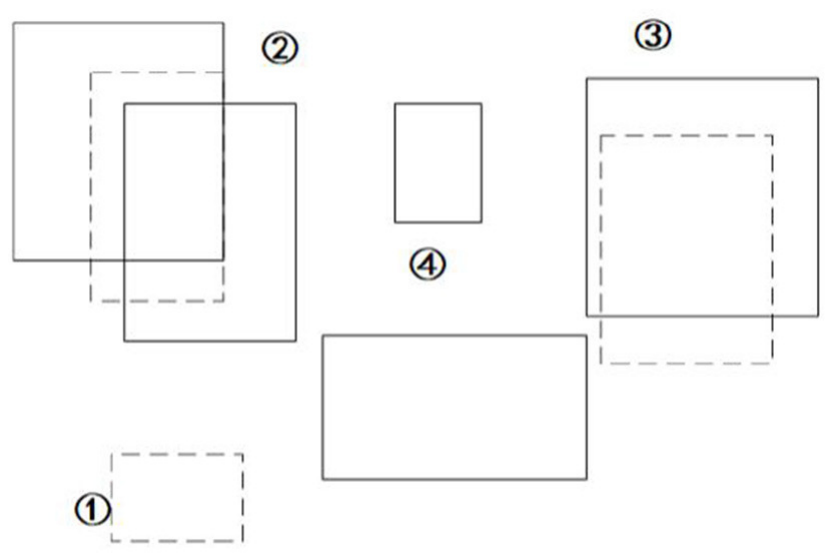
Schematic diagram of dynamic area detection.

In VSLAM, to ensure the accuracy of map point construction, it is necessary to eliminate all possible dynamic targets in the keyframe by comparing the information of the last frame when generating the keyframe. Due to the instability of the feature points, the calculated affine matrix has errors, so when the dynamic target is moving, the real-time calculation result using the frame difference method is often not satisfactory. Our method in this paper establishes dynamic candidate areas through keyframes. At the same time, it employs the YOLOv3 algorithm to receive each candidate area and discard candidate areas that cannot be identified. The method employs the GMM model to train the background image, estimates the motion area when creating new keyframes, provides prior knowledge for YOLOv3, and exploits the fast and robust advantages of YOLOv3 to achieve dynamic target detection between consecutive frames. With the advantage of discontinuous VSLAM keyframes in time series, each time a keyframe is established, this method analyzes the dynamic area to increase or decrease the dynamic tracking frame. This method can ensure the real-time performance of VSLAM and avoid the problem of local map tracking failure caused by too few map points due to multiple additions and reductions of candidate areas. The schematic diagram is shown in Figure 6.

The dotted box represents the dynamic candidate area provided by GMM, and the solid box represents all targets detected by YOLOv3. Then we use the IOU result as the probability information to get the largest possible dynamic target [such as at ②], discard the area where GMM dynamic detection fails [such as ①], discard other static targets obtained by YOLOv3 [such as ④], and detect the area (solid box) such as ②③.

### 3.2. Dynamic target tracking based on an improved Kalman filter

Multi-target detection algorithms are easily affected by factors such as illumination, occlusion, and pixel blur when moving (Li and Shi, 2019). The dynamic area will not disappear irregularly, so we build a tracking model to achieve multi-target tracking between two keyframes to ensure the continuity of the bounding box detected by YOLO. The Kalman filter algorithm itself is a linear system. Since the value observed in this paper is the state value, it is easy to estimate the value from the previous state to the next state by using the state transition matrix of the Kalman filter algorithm. The Kalman filter algorithm only considers the relationship between the upper and lower frames to a certain extent, so the Kalman filter algorithm needs a very accurate observation effect. However, in the actual operation process, the observed values are not necessarily accurate, whether due to the influence of the camera or lighting effects. To solve such problems, we propose an improved Kalman filter method. We exploit the improved Kalman filter to predict the maximum probability position and length and width information of the next frame. It uses its error covariance to calculate the predicted value of the state variable, find the observed value by combining the detection algorithm, correct the predicted value with Kalman gain, and finally obtain the optimal value of the variable.

**Figure 7:**
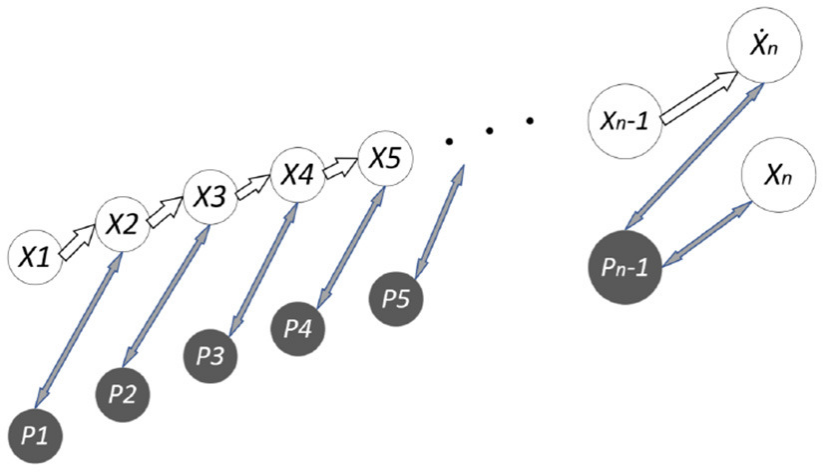
Improved observation value selection principle diagram.

The improved Kalman filtering algorithm exploits the first N groups of observations to establish a nonlinear fitting curve to predict the next group of observations. The algorithm uses an evaluation index to determine the selected predicted “observed value” or the value observed by the system. Since the feature points are affected by environmental factors or camera shake factors, linear fitting is performed according to the absolute values of the errors of the previous N-1 groups of predictions and observations. While ensuring the real-time performance of the algorithm, it can distinguish whether the target is moving fast or instantaneously due to observation errors. The improvement principle is shown in Figure 7 (*Ẋ*_*n*_ are fitted observations, and *X*_*n*_ is an actual observation. *P*_1_, *P*_2_… *P*_*n*−1_ represent the error covariance).

The observation values selected in this paper are the central pixels $\left(\frac{1}{2}\overset{2}{\underset{2i}{\sum }}x_{2i},\frac{1}{2}\overset{3}{\underset{2i+1}{\sum }}y_{2i+1}\right)$ of the four boundary corners of the target image to input to the Kalman filter system to obtain the predicted value. Then, take the predicted point as the center, use the value of max{|*x*_*j*_ − *x*_*i*_|} obtained in the last frame as the width of the rectangle, and the value of max{|*y*_*j*_ − *y*_*i*_|} as the length of the rectangle, and then crop a new area. The camera pose is detected and calculated in this area to obtain a new set of measured values, and the rectangular area size and area of the next frame are updated from the measured values and the new boundary corner points.

First, we establish an 8-dimensional state vector and a 4-dimensional observation vector according to the linear condition satisfied by the Kalman filter. The 8-dimensional state vector values represent the center pixel position x and y, the aspect ratio and height of the bounding box, and their corresponding velocity values. Expressed by the equation of motion *x*_*k*_ = *A*_*k*_*x*_*k*−1_ + *B*_*k*_μ_*k*_ + *w*_*k*_, due to the lack of a control vector, *B*_*k*_ is set to a 0 vector, which satisfies the state transition matrix:


(5) $$\begin{array}{l} A=\left[\begin{array}{cccccccc} 1 & 0 & 0 & 0 & 1 & 0 & 0 & 0\\ 0 & 1 & 0 & 0 & 0 & 1 & 0 & 0\\ 0 & 0 & 1 & 0 & 0 & 0 & 1 & 0\\ 0 & 0 & 0 & 1 & 0 & 0 & 0 & 1\\ 0 & 0 & 0 & 0 & 1 & 0 & 0 & 0\\ 0 & 0 & 0 & 0 & 0 & 1 & 0 & 0\\ 0 & 0 & 0 & 0 & 0 & 0 & 1 & 0\\ 0 & 0 & 0 & 0 & 0 & 0 & 0 & 1 \end{array}\right] \end{array}$$

This formula expresses the displacement of the previous state plus the unit velocity to represent the displacement of the current state, and considers the system error and the observation error, *w*_*k*_ ~ *N*(0, *Q*_*k*_), *v*_*k*_ ~ *N*(0, *R*_*k*_). The observation equation is expressed as *z*_*k*_ = *H*_*k*_*x*_*k*_ + *v*_*k*_ according to the Kalman filter. Because of the special relationship between the observation equation and the state equation in this paper, *H*_*k*_ is a 4×8 matrix, where the observation equation is only related to the first four dimensions of the current state vector, that is, the displacement point, so take:


(6) $$\begin{array}{l} H_{k}=\left[\begin{array}{cccccccc} 1 & 0 & 0 & 0 & 0 & 0 & 0 & 0\\ 0 & 1 & 0 & 0 & 0 & 0 & 0 & 0\\ 0 & 0 & 1 & 0 & 0 & 0 & 0 & 0\\ 0 & 0 & 0 & 1 & 0 & 0 & 0 & 0 \end{array}\right] \end{array}$$

To satisfy the system's optimal estimation of the state equation, we modify the state value during the observation phase and introduce the covariance matrix to update:


(7) $$\begin{array}{l} P_{k|k-1}=A\cdot P_{k-1|k-1}\cdot A^{T}+Q \end{array}$$

where *P*_*k*∣*k*−1_ represents the covariance matrix of the predicted state value and obtains the optimal estimation of the current state through the prediction result of the current system and the measurement of the current state:


(8) $$\begin{array}{l} x_{k|k}=x_{k|k-1}+K_{k}\left(z_{k}-H\cdot x_{k|k-1}\right) \end{array}$$

where *K*_*k*_ represents the current Kalman gain coefficient, which is represented by the covariance matrix P and the measurement matrix H:


(9) $$\begin{array}{l} K_{k}=P_{k|k-1}\cdot \frac{H^{T}}{\left(H\cdot P_{k|k-1}\cdot H^{T}+R\right)} \end{array}$$

We bring the Kalman gain at this time into the optimal estimation solution and exploit this gain to calculate the required covariance matrix value at the next moment:


(10) $$\begin{array}{l} P_{k|k}=\left(1-K_{k}\cdot H\right)P_{k|k-1} \end{array}$$

We can completely predict the center point's position at the next moment through observations. However, the selection of observations affects the stability of the entire system. In the case of minimal noise, if there is a significant error in the observed value, the predicted value will also be inaccurate. We improve the performance of the entire system by improving the selection of observations, and the method is as follows:

(1) Initialization phase

First, we establish a non-linear loss function model. Our method sets the model as:


(11) $$\begin{array}{l} f\left(x\right)-exp\left(a\cdot x^{2}+b\cdot x+c\right) \end{array}$$

Secondly, we define *N* groups of observation data (*N* is set to 20 in this chapter), and establish the least square function through the observation data:


(12) $$\begin{array}{l} \text{again}\underset{x}{\sum }||f\left(x\right)-\text{exp}\left(a\cdot x^{2}+b\cdot x+c\right)|{|}_{2} \end{array}$$

At the same time, we assign values to the initial values of the first N groups. If all the first N groups are assigned a value of 0, the finally obtained parameters are easy to fall into the local optimal solution, and the parameters to be sought are solved incorrectly in the initialization stage. Therefore, we add Gaussian disturbance to the value of f(x) and x to make them in a fluctuating state.

(2) Solving stage

To ensure that the data of a given fitting does not increase over time, the problem of incorrect fitting parameters and a significant increase in the number of calculations does not occur. Our method accepts new data while removing the old data to maintain it at the value of *N*. Within the parameter range. Our method uses the L-M method to iterate, and finally finds the solution of the unit at the next time through the known parameters, which is the “observation point” for solving the prediction.

(3) Judgment stage

Our method has obtained two sets of observation points: the observed points and the predicted “observation points.” Of course, it is hoped that the actual observation points are accurate, but regardless of the presence of noise or the influence of light factors, the observed data may always be wrong. This paper introduces third-party evaluation indicators to determine which value is more accurate.

**Figure 8:**
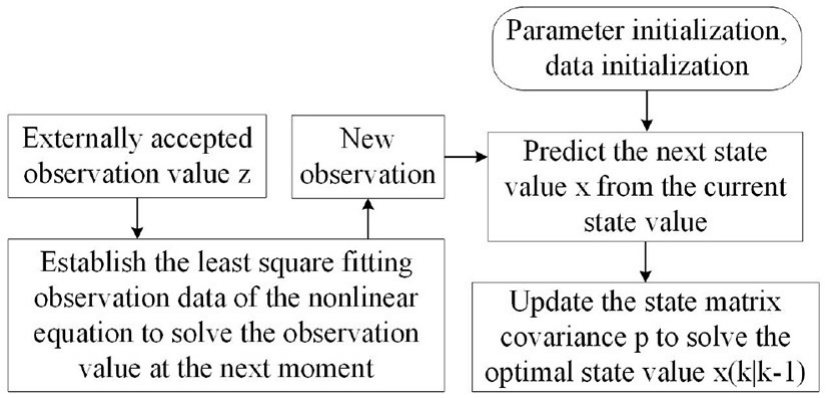
Improved Kalman forecast update flow chart.

We assume that the previous observation data are accurate (or the observation data has been corrected), and there are also errors between the predicted value of the Kalman filter and the observation of the next frame, and the error may be small. Our method builds a set of fitting data by the absolute value of the error between the observation value of the next frame and the predicted value of Kalman filter. At the same time, we fit the linear equations with the previous N-1 sets of data, predict the “observed value” of the Nth set of data, and calculate the absolute value of the error between it and the Kalman predicted value. Finally, we judge which observation value is more reliable according to the error growth rate. The calculation function of the judgment is as follows:


(13) $$z_{k}=\underset{z_{k}}{min}\{3\times ||\hat{z_{k}}-p_{k-1}|-g_{k}|\},\{||z_{k}-p_{k-1}|-g_{k}|\}$$

where *g*_*k*_ is the predicted value of the error, $\hat{z_{k}}$ is the predicted “observed value,” *z*_*k*_ is the observation value of the system, and *p*_*k*−1_ is the predicted value of the last frame. In order to ensure the reliability of system prediction, we assign weight to both of them to avoid local optimization. Finally, the closer “observation point” is selected as the new observation point. The flow chart of the algorithm is shown in Figure 8.

### 3.3. Incremental model

**Figure 9:**
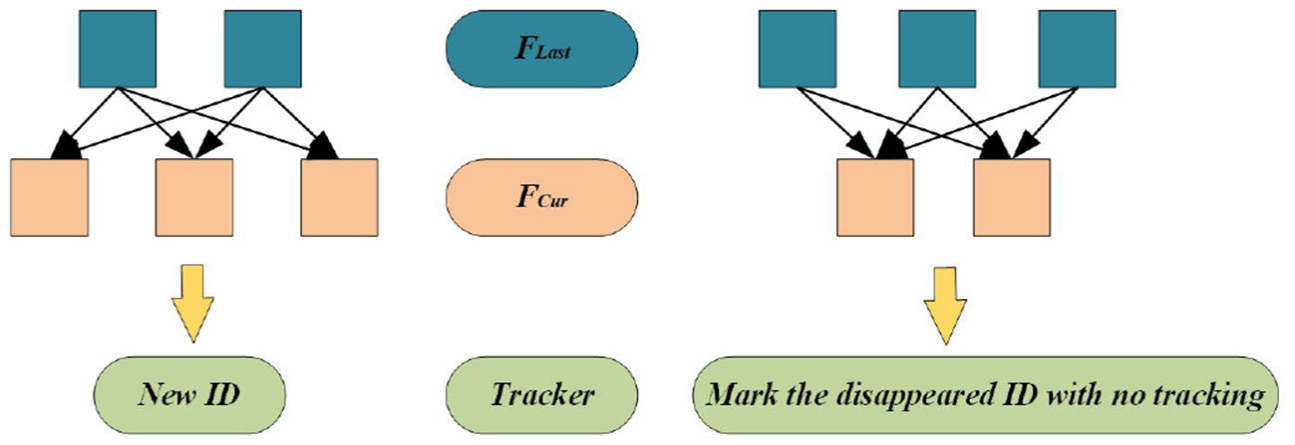
Incremental model.

The method provided in this paper performs the above dynamic target detection and tracking on the image sequence between every two keyframes. When VSLAM constructs a keyframe, it rejudges whether a new target area needs to be constructed. Therefore, the following incremental model is added during the keyframe construction to ensure that the dynamic increment can be tracked stably in the tracking thread or use the incremental model to determine whether to cancel tracking the lost target information. The incremental model is shown in Figure 9, where *F*_*L*ast_ represents the last frame of the keyframe, *F*_*C*ur_ represents the current frame that can also be understood as a keyframe, and *Tracker* is the tracker designed in this paper.

## 4. Experiments

This experiment utilizes the dynamic objects dataset in the TUM dataset for dynamic target detection and the verification of the tracking algorithm based on the improved Kalman filter. Finally, the algorithm is integrated into the VSLAM to eliminate the dynamic target. We verify the effectiveness of the algorithm proposed in this paper by two metrics: ATE (absolute trajectory error) and RPE (relative pose error). The test platform for this experiment is Ubuntu 16.04, the primary language for building the platform is C++, and the Python environment is applied for ATE and RPE analysis.

### 4.1. Analysis of target detection results based on the dynamic environment

**Figure 10:**
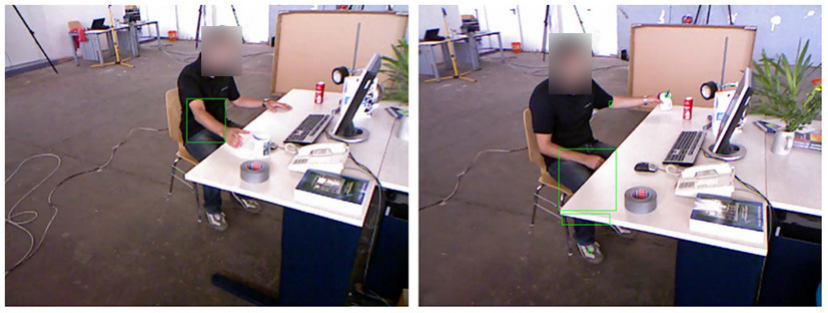
Dynamic detection performance effect of the data set.

At present, there is no clear data set for dynamic target detection in a dynamic environment. In order to verify the robustness of the dynamic target detection algorithm proposed in this paper, we search for dynamic targets in the Dynamic Object dataset in the TUM dataset. First, we employ YOLOv3 to set prior knowledge to label dynamic targets artificially. Next, we find the IOU value of the target detected by YOLOv3 and the result of dynamic target detection. The larger the experimental result, the more concentrated the detection distribution and the higher the detection accuracy. In order to reflect the superiority of the detection algorithm proposed in this paper, this experiment employs the traditional frame difference method, optical flow method and other algorithms that are often used in dynamic target detection to compare. Table 1 shows the results (calculate the average IOU value for each frame detected under each data set). For multiple dynamic targets in an image frame, calculate the average value of IOU in the current frame and then map it to the global data set. The algorithm's performance in this paper on the data set fr2/desk_with_person is shown in Figure 10.

**Table 1:** Dynamic target detection results.

**Data sets**	**Temporal difference method**	**Optical flow method**	**Ours**
fr2/desk_with_person	0.2514	0.4613	**0.5756**
fr3/sitting_static	0.1011	0.2167	**0.4783**
fr3/sitting_xyz	0.2331	0.5098	**0.5933**
fr3/sitting_halfsphere	0.4060	0.4200	**0.5749**
fr3/sitting_rpy	0.1991	0.2340	**0.6764**
fr3/warking_static	0.4788	0.6993	**0.6032**
fr3/warking_xyz	0.5423	0.5745	**0.7220**
fr3/warking_halfsphere	0.4421	0.6421	**0.6854**
fr3/warking_rpy	0.5322	0.3210	**0.6010**

Bold values mean the best result among the methods.

It can be seen from the data in Table 1 that the target detection algorithm used in this paper effectively improves the detection accuracy of the dynamic region.

### 4.2. Analysis of long-term tracking results based on YOLOv3 and improved Kalman filter

**Figure 11:**
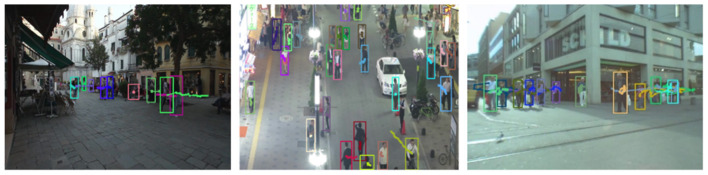
Operation effect of the tracking algorithm MOT16 in this paper.

This experiment utilizes the Dynamic Object dataset to verify the effectiveness of target tracking, and utilizes the MOT16 dataset to verify the robustness of the multi-target tracking algorithm used in this paper. This experiment utilizes YOLOv3 to detect pedestrians, and utilizes an improved Kalman filter algorithm to track the observation results provided by YOLOv3. We employ the Hungarian algorithm to find the match between the previous and next frames in terms of data association. The effect of running on MOT16 is shown in Figure 11.

The experimental results show that in a highly dynamic environment, the detection and tracking algorithm can better assign weights and find the best prediction results. It assigns the maximum possible motion trajectory to the target through cascade matching, avoiding the problem of target loss caused by occlusion.

### 4.3. Analysis of experimental results based on the VSLAM dynamic environment

**Figure 12:**
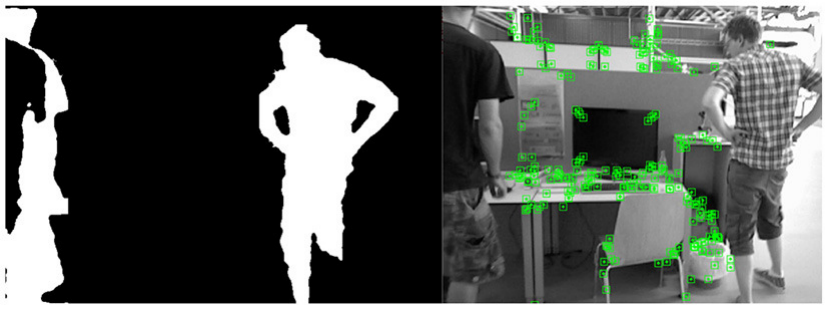
The feature collection effect of our method under the TUM data set fr3/w_xyz.

The segmentation idea we adopt is that under the target area frame, the proportion of target pixels is always the larger one, so we perform a sliding window search according to the depth value of the depth image to search for the pixel area with the largest proportion (we divide the depth image pixels into 16. There are 16-pixel areas per copy to ensure that each pixel value from 0 to 255 can be searched). In the augmented reality technology, the reason for the deviation of the virtual object in the map is often the calculation error of the posture point. Therefore, we use two indicators, ATE and RPE, to verify the algorithm in this paper. At the same time, in order to ensure that the method can be effectively applied to the augmented reality environment, we exploit the TUM data set fr3/w_xyz combined with the Augmented Reality Registration algorithm for verification. The feature collection effect of our method under the TUM data set fr3/w_xyz is shown in Figure 12. The binary image on the left is the result of dynamic target segmentation, and the image on the right is the feature points detected by VSLAM.

**Figure 13:**
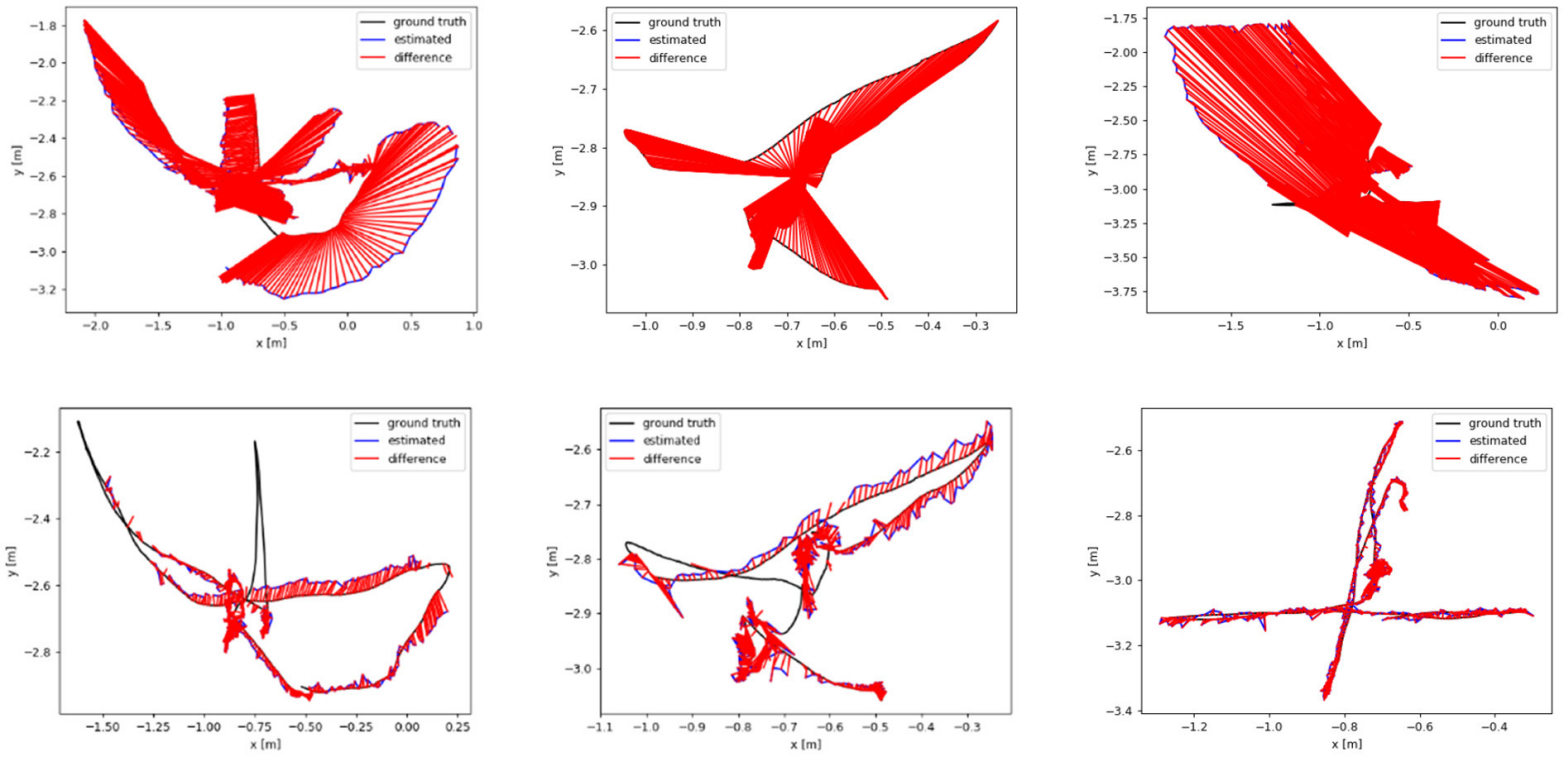
The **(top)** row is the trajectory error graph of ORB-SLAM2, and the **(bottom)** row is the trajectory error graph of ours. The ground truth, the estimated camera motion, and the localization error for each camera pose are represented as the black, blue, and red segments, respectively.

**Figure 14:**
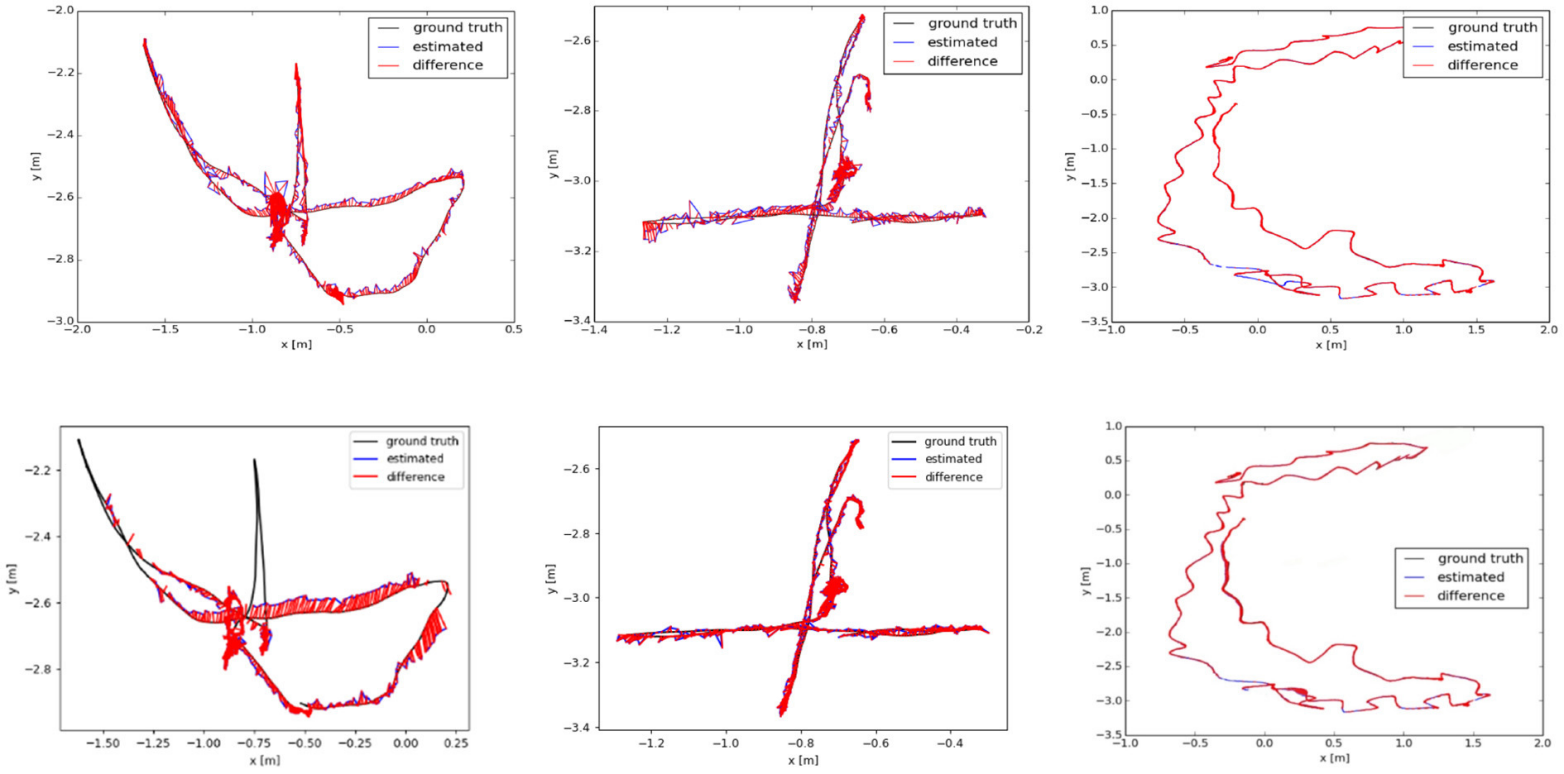
The **(top)** row is the trajectory error graph of SMR, and the **(bottom)** row is the trajectory error graph of ours. The ground truth, the estimated camera motion, and the localization error for each camera pose are represented as the black, blue, and red segments, respectively.

We analyze the results of multiple dynamic data sets in the TUM data set, and employ the absolute trajectory error graph ATE to verify the algorithm in this paper. It directly measures the point difference between the real trajectory and the estimated trajectory. The longer the red segment, the larger the estimation error and the lower the positioning accuracy. The ground truth, the estimated camera motion, and the localization error for each camera pose are represented as the black, blue, and red segments, respectively. The algorithm proposed in this paper is compared and analyzed with ORB-SLAM2 (Mur-Artal and Tardós, 2017) and SMR-SLAM (Cheng et al., 2019). Figure 13 shows the analysis results of the performance comparison between the proposed algorithm and ORB-SLAM2 under the conditions of three dynamic data sets fr3/w_half, fr3/w_rpy, and fr3/w_xyz. Figure 14 shows the analysis results of the performance comparison between the proposed algorithm and SMR-SLAM under the conditions of three dynamic data sets fr3/w_half, fr3/w_xyz, and fr2/desk_with_person.

Through the analysis of Figures 13, 14, it can be seen from the results of absolute trajectory error analysis that the algorithm proposed in this paper has more significant advantages in dynamic scenes and still maintains good results in low-dynamic scenes.

To reflect that the algorithm in this paper can maintain stable and superior performance under different data sets, we employ the official ATE and RPE test files provided by TUM to test the fr2 and fr3 series of data sets and obtain the data results shown in Tables 2–4. RMSE is the mean root mean square error, and SD is the standard deviation, using ORB-SLAM2 (RGB-D) (Mur-Artal and Tardós, 2017), MR-SLAM (Sun et al., 2018), and SMR-SLAM (Cheng et al., 2019) as comparisons.

**Table 2:** Ate in meters for the experiments using ORB-SLAM2, MR-SLAM, SMR-SLAM, and Ours.

**Data sets**	**ORB-SLAM2**		**MR-SLAM**		**SMR-SLAM**		**Ours**	
	**RMSE**	**S.D**.	**RMSE**	**S.D**.	**RMSE**	**S.D**.	**RMSE**	**S.D**.
w_halfsphere	0.2668	0.1429	0.0668	0.0266	0.0352	0.0207	**0.0342**	**0.0206**
w_xyz	0.2774	0.1230	01230	0.0657	**0.0186**	**0.0098**	0.0331	0.0176
w_rpy	0.1677	0.0958	0.0729	0.0335	0.0436	0.0253	**0.0347**	**0.0160**
w_static	0.0250	0.0147	0.0334	0.0207	0.0238	0.0113	**0.0142**	**0.0071**
s_halfsphere	0.0219	0.0133	0.0664	0.0386	**0.0210**	**0.0127**	0.0438	0.0305
s_xyz	**0.0089**	**0.0046**	0.0514	0.0280	0.0138	0.0076	0.0255	0.0113
desk_person	**0.0056**	**0.0030**	0.0759	0.0313	0.0068	0.0031	0.0728	0.0207

Bold values mean the best result among the methods.

**Table 3:** Translational drift (RPE) in m/s for the experiments using ORB-SLAM2, MR-SLAM, SMR-SLAM, and Ours.

**Data sets**	**ORB-SLAM2**		**MR-SLAM**		**SMR-SLAM**		**Ours**	
	**RMSE**	**S.D**.	**RMSE**	**S.D**.	**RMSE**	**S.D**.	**RMSE**	**S.D**.
w_halfsphere	0.8078	0.4958	0.0611	0.0268	0.0816	0.0419	**0.0539**	**0.0301**
w_xyz	0.6181	0.3778	0.0668	0.0369	**0.0337**	**0.0162**	0.0470	0.0227
w_rpy	1.5083	0.9031	0.0968	0.0510	0.0337	0.0162	**0.0214**	**0.0134**
w_static	0.5436	0.3783	0.0307	0.0205	0.0829	0.0479	**0.0276**	**0.0165**
s_halfsphere	0.0326	0.0198	0.0547	0.0318	**0.0307**	**0.0183**	0.0654	0.0429
s_xyz	**0.0132**	**0.0063**	0.0357	0.0225	0.0242	0.0106	0.0363	0.0167
desk_person	0.0383	0.0228	0.0213	0.0151	0.0369	**0.0213**	**0.0121**	0.0646

Bold values mean the best result among the methods.

**Table 4:** Rotational drift (RPE) in m/s for the experiments using ORB-SLAM2, MR-SLAM, SMR-SLAM, and Ours.

**Data sets**	**ORB-SLAM2**		**MR-SLAM**		**SMR-SLAM**		**Ours**	
	**RMSE**	**S.D**.	**RMSE**	**S.D**.	**RMSE**	**S.D**.	**RMSE**	**S.D**.
w_halfsphere	17.7267	10.2391	1.9004	0.7629	1.1556	0.5359	**1.0076**	**0.4283**
w_xyz	10.9428	7.1977	1.5950	0.8236	0.7473	0.4333	**0.7427**	**0.4266**
w_rpy	28.0287	17.3043	2.5936	1.3210	1.6024	0.9284	**1.0777**	**0.5112**
w_static	9.9384	6.9106	0.8998	0.6470	1.1366	0.6269	**0.4823**	**0.2975**
s_halfsphere	0.8217	0.3594	2.2677	1.3861	**0.8038**	**0.3495**	1.0254	0.4454
s_xyz	**0.5775**	0.3016	1.0362	0.5304	0.6905	0.3474	0.6601	**0.2998**
desk_person	1.4668	0.6857	**0.7744**	**0.4767**	1.3784	0.6742	1.4410	0.6932

Bold values mean the best result among the methods.

It can be seen from Tables 2–4 that the performance results of the VSLAM method proposed in this paper on the dynamic data set are much better than ORB-SLAM2. Compared with the more advanced VSLAM systems, MR-SLAM and SMR-SLAM, currently proposed, it also has an advantage. Although the performance on the low-dynamic dataset is slightly inferior to that of the SMR-SLAM algorithm, it still maintains a better advantage than ORB-SLAM2. This result is consistent with the results in Figures 13, 14. Our method occupies an absolute advantage in evaluating rotation drift, which can effectively avoid errors caused by dynamic target interference in many applications such as AR.

### 4.4. Experiments with augmented reality registration

#### 4.4.1. Robustness experiments

**Figure 15:**
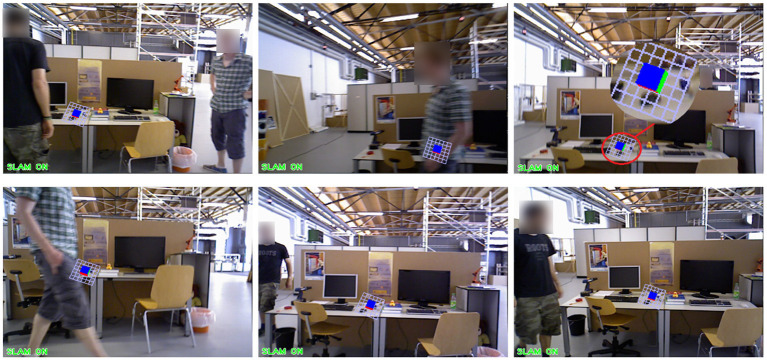
The application effect of ORB-SLAM2 method in augmented reality experiment.

**Figure 16:**
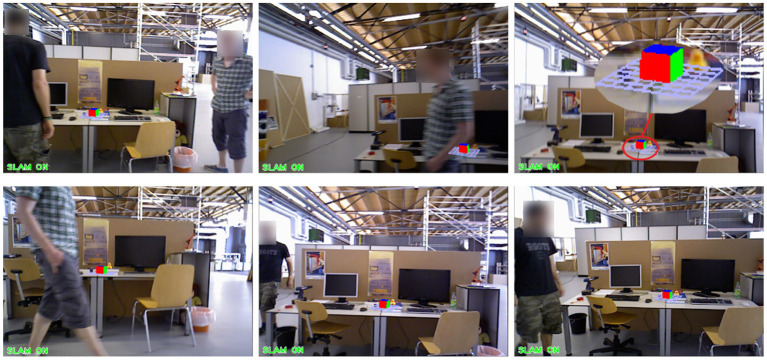
The application effect of our method in the augmented reality experiment.

The above experiment is the result analysis of the VSLAM algorithm we proposed under the dynamic data set. At the same time, in order to verify the robustness of our proposed method in the Augmented Reality system, we utilize the fr3/w_xyz data set to test, select the appropriate Kth frame, insert a virtual object, and observe the dynamic performance of the virtual object during operation. The experimental results of the ORB-SLAM2 method are shown in Figure 15. The experimental results of our proposed method are shown in Figure 16.

We choose to insert a virtual square in the 20th frame of the data set fr3/w_xyz. From frame 100 to frame 500, we sampled the result six times. In these six images, there are objects entering, a single object moving slowly, a single object moving quickly, multiple objects moving, the lens moving up and down, the lens moving left and right, and the lens rotating. Figures 15, 16 show the AR implementation effects of the ORB-SLAM2 method and the method in this paper, respectively. It can be seen from Figure 15 that under the influence of camera motion and video portrait motion, ORB-SLAM2 cannot accurately analyze the plane, and the error situation shown in Figure 15 often occurs. In terms of long-term attitude tracking, the ORB-SLAM2 method has attitude offset, which will also cause the inserted virtual object to not be in the original position. It can be seen from the results in Figure 16 that the VSLAM method proposed in this paper can accurately fit and create a virtual object, which greatly improves the registration of augmented reality and the tracking of virtual objects.

#### 4.4.2. Real-time experiment

We conduct real-time comparison experiments of Augmented Reality Registration in a dynamic laboratory environment. We register virtual objects at the 50th, 100th, 200th, 350th, and 500th frames after initialization, calculate the response time, and compare with our method through several classical algorithms such as SURF+KLT, ORB-SLAM2, and VINS-Mono (Mur-Artal and Tardós, 2017). The data are shown in Table 5. The experimental results show that the registration real-time performance of our method is better than the traditional SURF+KLT method at different time stages. Although, the computational cost of detection causes our method to consume slightly more time than the ORB-SLAM2 method for Augmented Reality Registration, this method provides better robustness while the registration latency remains stable below 25 ms.

**Table 5:** Real-time analysis (ms).

**Frame**	**SURF+KLT**	**VINS-Mono**	**ORB-SLAM2**	**Ours**
50	42.5	26.3	22.3	**23.9**
100	44.5	29.5	22.5	**23.1**
200	49.0	44.9	23.5	**24.5**
350	50.2	69.9	23.2	**24.9**
500	50.5	108.6	23.4	**24.8**

Bold values mean the best result among the methods.

#### 4.4.3. Comparison experiment with VISLAM

**Figure 17:**
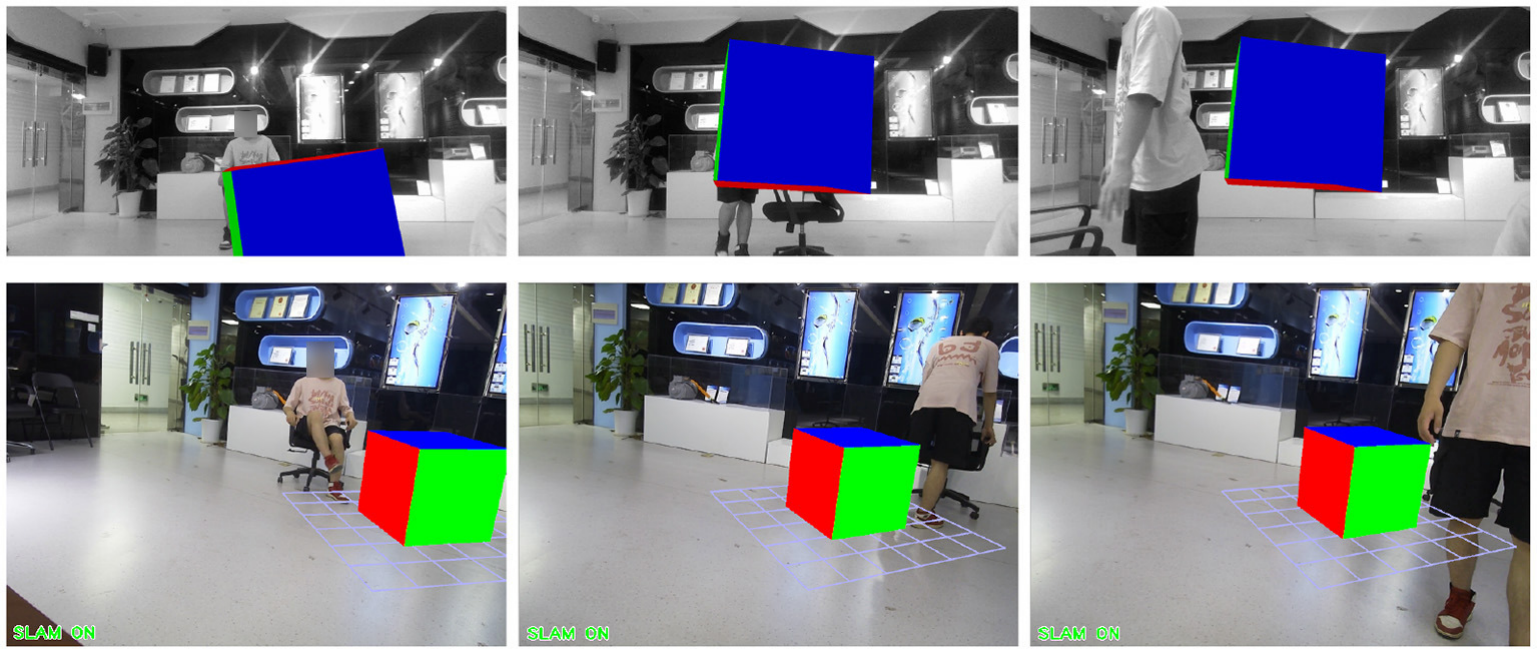
Application of VINS and our method in augmented reality experiment.

VISLAM is the most commonly used registration method for AR today. Although, the use of IMU provides good assistance for camera pose, it does not perform so well when tracking for long periods of time in dynamic environments. As shown in the VINS-Mono data in Table 5, after 350 frames, it shows a great drift and the registration time is also much longer. In the laboratory dynamic environment, we carried out many experiment of dragging the chair to move. After initialization, insert a virtual object, and verify by dragging the chair to move together. We select one of the experimental results for comparison, as shown in Figure 17. It can be seen from the results that the virtual objects registered by the VINS-Mono method are not very robust in dynamic environments. However, the virtual objects registered by our method remain stable in long-term dynamic environments.

## 5. Conclusion

In recent years, augmented reality technology is prevalent, and it is often applied in small map scenarios. Therefore, a small number of dynamic points in the map will significantly affect the registration effect. The dynamic target detection and tracking algorithm proposed in this paper can effectively help the stable operation of the Augmented Reality Registration technology in a dynamic environment. The stable operation of YOLOv3 can effectively help eliminate the feature points of small dynamic targets. Considering that the offset of augmented reality in the map is always the calculation error of the pose point is too large, this paper uses the ATE and RPE indicators to verify the algorithm of this paper. The final result analysis shows that the algorithm proposed in this paper has an excellent performance in each target detection stage and long-term tracking. The results of the ATE and RPE indicators indicate that the algorithm proposed in this paper performs well in both small and large dynamic scenarios and can be well applied in augmented reality technology. When we integrated the object detection method into the SLAM system, we did not choose the more efficient YOLOv4 due to the problem of computing power. Therefore, we use the prior data provided by GMM to compensate for the accuracy problem, which can use less computing power while maintaining the accuracy and real-time required for Augmented Reality Registration. There are better solutions now, like YOLOv5 and the recently released YOLOv7, and we're working on it. And, we need to optimize the computational cost in the next work so that Augmented Reality Registration requires less computational power and has better real-time performance.

## Data availability statement

The original contributions presented in the study are included in the article/supplementary material, further inquiries can be directed to the corresponding author/s.

## Author contributions

JL and DC provided research ideas and plans. JL and QG improved the algorithm. QG and DY wrote the programs and conducted the experiments. DC and QG were responsible for collecting data. QG wrote the manuscript with the help of JL and DC. DC revised the manuscript and approved the final submission. All authors contributed to the article and approved the submitted version.

## Funding

This work was partially supported by the Key R&D Program of Jiangsu Province (Industry Prospects and Key Core Technologies) under Grant BE2020006-2, the National Natural Science Foundation of China under Grants 61773219 and 62003169, the Natural Science Foundation of Jiangsu Province under Grant BK20200823, the Jiangsu Innovation and Entrepreneurship Talent Program Project under Grant JSSCBS202030576, and the Natural Science Research Project of Jiangsu Higher Education Institutions under Grant 20KJB520029.

## Conflict of interest

The authors declare that the research was conducted in the absence of any commercial or financial relationships that could be construed as a potential conflict of interest.

## Publisher's note

All claims expressed in this article are solely those of the authors and do not necessarily represent those of their affiliated organizations, or those of the publisher, the editors and the reviewers. Any product that may be evaluated in this article, or claim that may be made by its manufacturer, is not guaranteed or endorsed by the publisher.
